# Supplementary material for: MicroRNA-138-5p Targets Pro-Apoptotic Factors and Favors Neural Cell Survival: Analysis in the Injured Spinal Cord
Source: Biomedicines. 2022 Jun 30;10(7):1559. doi: 10.3390/biomedicines10071559 (PMC9312482; doi:10.3390/biomedicines10071559)
Supplement: Supplementary file 1 [file biomedicines-10-01559-s001.zip › Figure S1.pdf]

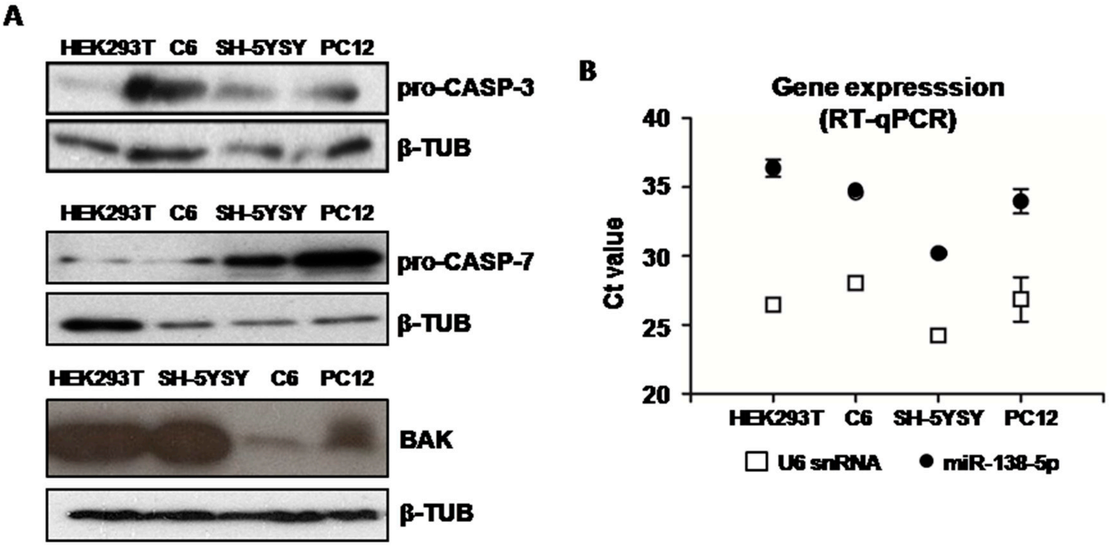

Figure S1. Endogenous expression of miR-138-5p and its targets in different cell lines. (A) Endogenous. expression of pro-CASP-3, pro-CASP-7 and BAK proteins, and (B) miR-138-5p microRNA in HEK293T, C6, SH-SY5Y and PC12 cell lines. MicroRNA expression was measured by RT-qPCR, whereas the protein expression of its targets was estimated by immunoblot (the image shown is a representative example). Complete blot images are available at OSF (https://osf.io/yqn58/). All experiments were carried out in triplicates.
