# Supplementary material for: MicroRNA-138-5p Targets Pro-Apoptotic Factors and Favors Neural Cell Survival: Analysis in the Injured Spinal Cord
Source: Biomedicines. 2022 Jun 30;10(7):1559. doi: 10.3390/biomedicines10071559 (PMC9312482; doi:10.3390/biomedicines10071559)
Supplement: Supplementary file 1 [file biomedicines-10-01559-s001.zip › Figure S2.pdf]

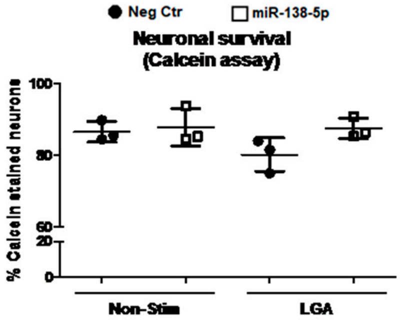

Calcein (Three-way ANOVA)					
Calcein~EXP+MIR*LGA					
	Df	Sum Sq	Mean Sq	F value	Pr (>F)
EXP	2	89.36	44.68	6.630	0.0302 *
MIR	1	55.79	55.79	8.279	0.0282 *
LGA	1	34.08	34.08	5.057	0.0656
MIR:LGA	1	27.70	27.70	4.110	0.0890
Residuals	6	40.44	6.74		

Figure S2. MiR-138-5p induces neuronal survival after LGA treatment. Neuronal survival (% of calcein-AM stained cells) was analyzed in hippocampal neuron cultures transfected with either miR-138-5p (white squares) or negative control (Neg Ctr; black circles) mimics followed by LGA stimulation. Dot plots represent the average ± SD of three independent experiments. Table details the results from the three-way ANOVA analyses. EXP codes for the experiment block factor; MIR for mir-138-5p transfection; and LGA for LGA stimulation.
