# Supplementary material for: MicroRNA-138-5p Targets Pro-Apoptotic Factors and Favors Neural Cell Survival: Analysis in the Injured Spinal Cord
Source: Biomedicines. 2022 Jun 30;10(7):1559. doi: 10.3390/biomedicines10071559 (PMC9312482; doi:10.3390/biomedicines10071559)
Supplement: Supplementary file 1 [file biomedicines-10-01559-s001.zip › Figure S3.pdf]

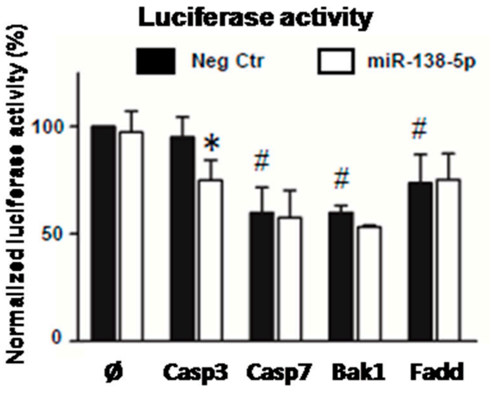

Figure S3. MiR-138-5p targets the 3'UTR of Casp3, Casp7 and Bak1 genes on HEK293T. Human HEK293T cell cultures were employed to measure the luciferase activity of the 3'UTR reporter constructs of the Casp3, Casp7, Bak1, Fadd and the empty construct (Ø; pmirGLO without 3'UTR subcloned). Cell cultures were transiently cotransfected with control or reporter constructs and miR-138-5p (white bars) or the negative control mimics (black bars). Bars represent the mean ± SD of at least three independent experiments. Luciferase activity of the different 3'UTR reporter constructs co-transfected with the negative control mimic (Neg Ctr) was compared through a paired t-test (# $p<0.05$, relative to control construct). The effect of transfecting miR-138-5p mimic relative to the negative control mimic (Neg Ctr) in the luciferase activity ratio of each co-transfected 3'UTR reporter construct was analysed using a paired t-test. * denotes a significant difference ($p<0.05$) relative to its corresponding negative control.
