# Supplementary material for: MicroRNA-138-5p Targets Pro-Apoptotic Factors and Favors Neural Cell Survival: Analysis in the Injured Spinal Cord
Source: Biomedicines. 2022 Jun 30;10(7):1559. doi: 10.3390/biomedicines10071559 (PMC9312482; doi:10.3390/biomedicines10071559)
Supplement: Supplementary file 1 [file biomedicines-10-01559-s001.zip › Figure S4.pdf]

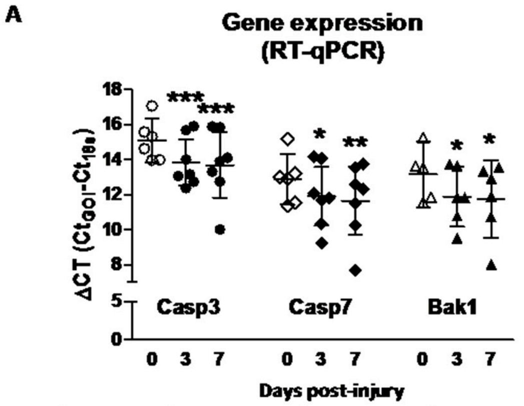

Casp3	0 dpi	3 dpi	7 dpi
mean ΔCt	15.08 ± 1.17	13.84 ± 1.42	13.68 ± 2.01
$2^{-\Delta\Delta Ct}$ (95% CI)	1.00 (0.41 ; 2.42)	2.37 (0.68 ; 8.23)	2.64 (0.68 ; 10.29)

Kruskal Wallis	df	χ^2 value	p-value	Conover test p-value		
	2	12.051	0.002		3dpi-0dpi	0.0003
					7dpi-0dpi	0.0004
					7dpi-3dpi	0.728

Casp7	0 dpi	3 dpi	7 dpi
mean ΔCt	12.86 ± 1.37	11.90 ± 1.80	11.66 ± 2.11
$2^{-\Delta\Delta Ct}$ (95% CI)	1.00 (0.40 ; 2.47)	1.94 (0.63 ; 5.91)	2.30 (0.62 ; 8.54)

ANOVA	df	F value	p-value	Tukey test p-value		
Exp	6	15.822	0.00007		3dpi-0dpi	0.024
dpi	2	8.287	0.006		7dpi-0dpi	0.008
					7dpi-3dpi	1.000

Bak1	0 dpi	3 dpi	7 dpi
mean ΔCt	13.15 ± 1.50	11.88 ± 1.63	11.74 ± 2.10
$2^{-\Delta\Delta Ct}$ (95% CI)	1.00 (0.33 ; 3.04)	2.40 (0.67 ; 8.52)	2.65 (0.56 ; 12.62)

ANOVA	df	F value	p-value	Tukey test p-value		
Exp	5	11.793	0.0009		3dpi-0dpi	0.025
dpi	2	7.914	0.010		7dpi-0dpi	0.018
					7dpi-3dpi	1.000

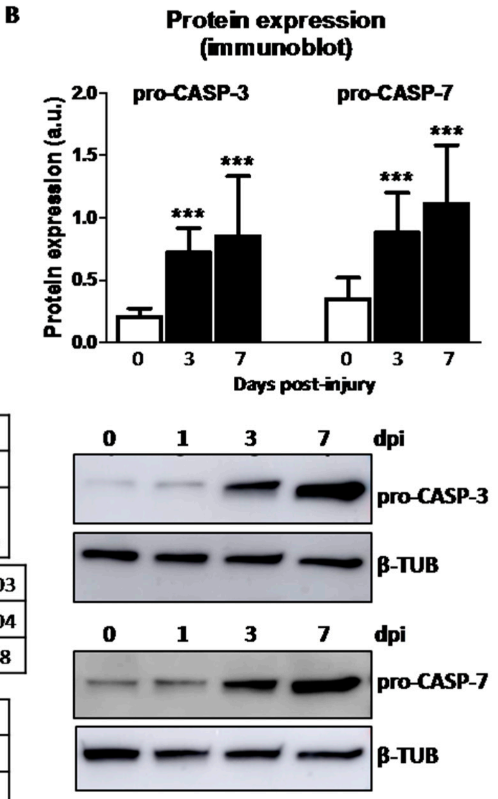

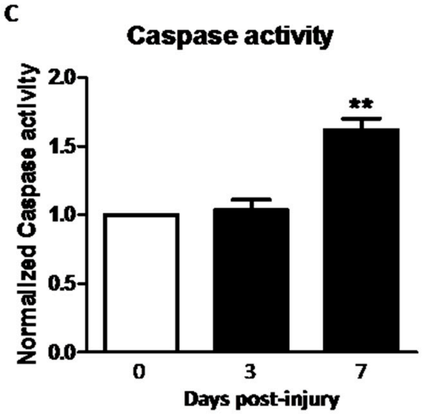

Figure S4. Expression and activity of caspase 3, caspase 7 and BAK1 increases at 3 and 7 days after SCI. (A) RT-qPCR showing relative Casp3, Casp7 and Bak1 expressions in RNA isolated from spinal cord samples collected at 0, 3 and 7 dpi. Dot plots represent mRNA expression as the average of ΔCt ± 95% CI. Tables show the mRNA expression of Casp3, Casp7 and Bak1 as the mean ΔCt and the fold change $2^{-\Delta\Delta Ct}$ (values and limits in folds of the 95% CI) from, at least, five individuals per time. Statistical analysis was carried out through Kruskal-Wallis test or two-way ANOVA and Tukey post-hoc tests. (B) Protein expression of pro-CASP-3 and pro-CASP-7 at 0, 3 and 7 dpi. The bar graph shows the densitometry data of each apoptotic protein after SCI. Bars correspond to the average ± SD of six animals. Statistical analysis was carried out through Kruskal-Wallis and Conover post-hoc tests. Complete blot images are available as supplementary material (https://osf.io/yqn58/). (C) Effector caspase activity of samples of 0, 3 and 7 dpi rats. Bars represent the average ± SD of four animals. Statistical significance of the changes was carried out through one-way ANOVA and Tukey post-hoc tests. *, ** and *** denote significant differences (p<0.05, p<0.01 and p<0.001, respectively) relative to control animals.
