# Supplementary material for: MicroRNA-138-5p Targets Pro-Apoptotic Factors and Favors Neural Cell Survival: Analysis in the Injured Spinal Cord
Source: Biomedicines. 2022 Jun 30;10(7):1559. doi: 10.3390/biomedicines10071559 (PMC9312482; doi:10.3390/biomedicines10071559)
Supplement: Supplementary file 1 [file biomedicines-10-01559-s001.zip › Table S1.pdf]

A

Probes	Sequence
miR-138-5p	5'-(C)GG(C)CU(G)AU(T)UA(T)AA(C)AC(C)AG(CT)-3'
cel-miR-67 (Neg. Ctr.)	5'-G(T)GU(A)AC(A)CG(T)CU(A)UA(C)GC(C)CA-3'

B

Immunoblotting antibodies	Comercial reference and antibody registry ID	Dilution
Rabbit anti-CASP-3	Cell Signaling Technology Cat#9662, RRID:AB_10694681	1:1000
Rabbit anti-CASP-7	Cell Signaling Technology Cat#9492, RRID:AB_10831368	1:1000
Rabbit anti-BAK	Cell Signaling Technology Cat#3814, RRID:AB_2290287	1:1000
Mouse anti -beta-tubulin	Sigma-Aldrich Cat#T5293, RRID:AB_477580	1:10000
HRP-conjugated goat anti-rabbit	Thermo Fisher Scientific Cat#31460, RRID:AB_228341	1:1000
HRP-conjugated goat anti-mouse	Thermo Fisher Scientific Cat#31430, RRID:AB_228307	1:1000
**Immunofluorescence antibodies**	**Comercial reference and antibody registry ID**	**Dilution**
Mouse anti-Glial Fibrillary Acidic Protein (GFAP), clone GA5	Millipore Cat#MAB3402, RRID:AB_94844	1:250
Rabbit anti-NeuN	Millipore Cat#ABN78, RRID:AB_10807945	1:250
Mouse anti-Adenomatous Polyposis Coli (Ab-7) (APC)	Millipore Cat#OP80, RRID:AB_2057371	1:250
Mouse anti-Tubulin, beta III isoform, C-terminus, clone TU-20	Millipore Cat#MAB1637, RRID:AB_2210524	1:500
Rabbit anti-CASP-3	Cell Signaling Technology Cat#9662, RRID:AB_10694681	1:300
Rabbit anti-CASP-7	Cell Signaling Technology Cat#9492, RRID:AB_10831368	1:100
Alexa 594-conjugated goat anti-mouse IgG2a	Innovative Research Cat#A21135, RRID:AB_1500827	1:500
Alexa 488-conjugated goat anti-mouse IgG (H+L) highly cross-adsorbed antibody	Molecular Probes Cat#A-11029, RRID:AB_138404	1:250
Alkaline phosphatase conjugated sheep-anti-digoxigenin	Roche Cat#11093274910, RRID:AB_514497	1:400

C

Primer	Sequence
Casp3 SacI-sense	5'-CCGGAGCTCTTGGGAGTGGGGTAGG-3'
Casp3 SalI-antisense	5'-CTTGTCGACATTGTCACATGGGAACAC-3'
Casp3-Mut138 NheI-sense	5'-TAATTTTACGCTAGCAAAATATC-3'
Casp3-Mut138 NheI-antisense	5'-GATATTTTGCTAGCGTAAAATT-3'
Casp7 SacI-sense	5'-ACTGAGCTCCTGCTGTCATTCACTGATG-3'
Casp7 SalI-antisense	5'-GTCGTCGACTTATTAGGAGGGGAAAACAGG-3'
Casp7-Mut138 NheI-sense	5'-GCCCATATCGCTAGCCGCCTTACC-3'
Casp7-Mut138 NheI-antisense	5'-GGTAAGGCGGCTAGCGATATGGGC-3'
Bak1 NheI-sense	5'-GCTAGCGAGCTCCAGCCACATGACTGC-3'
Bak1 SalI-antisense	5'-TGCGTCGACTATTTCCAGACACCGAGG-3'
Bak1-Mut138 EcoR1-sense	5'-CGCTAGAGAACTGAATTCAAAAGCAAC-3'
Bak1-Mut138 EcoR1-antisense	5'-GTTGCTTTTGAATTCAGTTCTCTAGCG-3'
Fadd SacI-sense	5'-GAGCTCTGCTGTGGAGGGAGCACC-3'
Fadd SalI-antisense	5'-GTCGACAAGAAATGGTTTATTTGGCCG-3'

Table S1. Probes, antibodies and primers employed in this study. (A) Sequences of the oligonucleotide probes employed for FISH analysis of miR-138-5p and the negative control. FISH probes are composed of 2′ -O-methyl and LNA (marked as () in the sequence) nucleotides according to the designs of Søe and cols.[27]. (B) Primary and secondary antibodies used for immunoblotting and immunofluorescence. Antibody registry ID corresponds to the unique identification of each antibody assigned by The Antibody Registry webpage (https://antibodyregistry.org/). (C) PCR primers employed in the subcloning of the 3' UTR regions of Casp3, Casp7, Bak1 and Fadd.
